# Playground Inclusivity for Children With a Disability: Protocol for a Scoping Review

**DOI:** 10.2196/37312

**Published:** 2022-07-22

**Authors:** Leah G Taylor, Leigh M Vanderloo, Kelly P Arbour-Nicitopoulos, Jennifer Leo, Jason Gilliland, Patricia Tucker

**Affiliations:** 1 Department of Health and Rehabilitation Sciences Western University London, ON Canada; 2 School of Occupational Therapy Western University London, ON Canada; 3 ParticipACTION Toronto, ON Canada; 4 Mental Health and Physical Activity Research Centre Faculty of Kinesiology and Physical Education University of Toronto Toronto, ON Canada; 5 Steadward Centre for Personal and Physical Achievement Faculty of Kinesiology, Sport, and Recreation University of Alberta Edmonton, AB Canada; 6 Department of Geography and Environment Faculty of Social Sciences Western University London, ON Canada; 7 Department of Paediatrics Schulich School of Medicine Western University London, ON Canada; 8 Department of Epidemiology and Biostatistics Schulich School of Medicine Western University London, ON Canada; 9 Children’s Health Research Institute Lawson Health Research Institute London, ON Canada

**Keywords:** playground, inclusion, children, disability, scoping review, protocol, youth, inclusivity, participation, young, accessibility, design, structure, application

## Abstract

**Background:**

Although playgrounds are designed to promote outdoor play, children with disabilities may be unable to engage in these spaces due to intrinsic and extrinsic factors. Previous research has examined inclusive/accessible playground design when developing new playgrounds; however, it is unclear if there is a best-practice tool for evaluating the inclusivity of existing playground structures.

**Objective:**

A scoping review of both peer-reviewed and grey literature will be employed to explore evaluation tools for playground inclusivity, to enable the participation of children with disabilities.

**Methods:**

The conduct of this study will adhere to the PRISMA-ScR (Preferred Reporting Items for Systematic Reviews and Meta-Analyses extension for Scoping Reviews) guidelines. A search for peer-reviewed research studies will be conducted in the following electronic databases: MEDLINE, Scopus, CINAHL, and Embase. Grey literature will be examined via a three-step process: (1) a search in the Canadian Health Research Collection Database; (2) a targeted Google search; and (3) reference list searching. Titles, abstracts, keywords, and full texts of identified studies will be independently screened for inclusion by two reviewers. A synthesis of included articles will describe the publication and auditing tool details. A summary of the findings will highlight the types of playgrounds measured, types of disability considered, measures of inclusion used, and psychometric properties.

**Results:**

Database searches for peer-reviewed articles were completed in December 2021. A total of 1471 unique records were returned after the removal of 559 duplicate records. Full texts of 167 studies meeting eligibility criteria will be reviewed. The peer-reviewed research search will guide the grey literature search. The scoping review is planned for completion in 2022.

**Conclusions:**

A rigorous search of the literature will determine the availability of tools for evaluating existing playground structures for the inclusivity of children with disabilities. The results will inform recommendations on tool applications, and applicable knowledge translation activities.

**International Registered Report Identifier (IRRID):**

DERR1-10.2196/37312

## Introduction

### Background

Play is an internationally recognized, fundamental experience of childhood. The United Nations (1989) [1] emphasized play as a priority in the *Convention on the Rights of a Child,* indicating that every child has the right to participate in age-appropriate play and recreation. Providing children with environments that support unstructured play opportunities is crucial to their physical, social, cognitive, and emotional development, and their long-term health and well-being [2,3]. Unstructured play is any self-chosen, immersive activity defined by the child, with no extrinsic goals, and undertaken for enjoyment [4]. Outdoor unstructured play offers opportunities for enhanced surroundings that facilitate sensory experiences, increase physical and social competencies, and promote fine and gross motor skill development [5]. When children play outside, they engage in rich, diverse, and active play [6]. Research in populations of typically developing children has shown outdoor unstructured play improves physical activity levels and cardiorespiratory fitness, and decreases sedentary behaviour, positively impacting children’s health [7-9].

Playgrounds are defined by the Canadian Standards Agency (2020) [10] as any fixed equipment used for play, typically found in parks, schoolyards, and childcare and recreation facilities. These environmental fixtures are designed to promote children’s outdoor, active, and imaginative play without financial barriers. Furthermore, playgrounds are an environmental context where children can develop social, physical, and motor skills through play and interaction with others [11,12]. Although access to playground infrastructure has been positively correlated with outdoor play for typically developing children [2], research suggests that children with disabilities do not share equal opportunities for accessing and engaging with playgrounds as their peers without disabilities [13], and face exclusion from community play spaces [14].

“Disability” is defined by the *United Nations Convention on the Rights of Persons with Disabilities* (2006) [15] as “physical, mental, intellectual or sensory impairments which, in interaction with various barriers, may hinder full and effective participation in society on an equal basis with others.” Recent research indicates an estimated 240 million children (under the age of 18 years) worldwide have one or more disabilities [16]. Children with disabilities may have unequal access to play opportunities due to such barriers (eg, restricted mobility, difficulty understanding play contexts or initiating/sustaining play with others [17]) that impede their full participation. In playgrounds, children’s play behaviours are determined by interactions with others and physical competence within the setting [18]. This means children who experience disabilities can experience exclusion from participation in play, due to factors such as inadequate access, inadequate play options and play value, and limited opportunities for social interaction [11].

Although outdoor play provides opportunities supportive of the health, development and well-being of all children, exclusionary environments limit the opportunities of children with disabilities to engage in these spaces [19]. Article 23 of the *Convention on the Rights of a Child* (1989) [1] indicates that children with disabilities “should enjoy a full and decent life, in conditions which ensure dignity, promote self-reliance and facilitate the child's active participation in the community.” Furthermore, “to achieve full inclusion, an accessible, barrier-free physical and social environment is necessary” [20]. A greater understanding of how to support and facilitate children’s interaction within playground environments is crucial to providing an inclusive experience and extending the benefits of participating in outdoor play.

Making an environment more “accessible” focuses on changing the design of the physical space to remove barriers that prevent full participation by people with disabilities. This is typically guided by local policy standards [21]. Making an environment accessible, however, does not make it *inclusive* [22]. Although “inclusion” encompasses accessibility, it is defined as the process of enabling the full participation of individuals with disabilities in activities, emphasizing the range of human diversity, to provide a space where all people belong [23]. Spencer-Cavaliere and Watkinson (2010) [24] indicate that there are three important aspects of inclusion for children with disabilities in the experience of play: (1) gaining entry to play, (2) feeling like a legitimate participant, and (3) having friends. Inclusion on the playground means creating environments where all children have equal access to opportunities for engaging in the physical and social aspects of play [25]. Therefore, targeting inclusion as an area for intervention, rather than accessibility alone, offers a more comprehensive approach that considers the subjective experience of children with disabilities and their families during playground visits [21,24].

Although installing new playgrounds that are inclusive for all users would be ideal, it is not realistic. Therefore, it is important to have access to tools to evaluate existing playground structures and set priorities for making changes/updates to improve inclusion. Playground audits are an example of a tool that can be used to measure and evaluate the detailed attributes of the play space environment [26,27]. Employing auditing tools to evaluate playground inclusion allows for investigation into the equity of the environment for children of all abilities, and the prioritization of necessary steps for action. Audits have been deemed important measures for evaluating the inclusion of children with disabilities in playground settings [28] and can be used in both research and practice [27]. However, it is unclear if a best-practice tool exists for evaluating the inclusivity of existing playgrounds. This warrants investigation, to bridge research with practice to offer tools and strategies that are usable for stakeholders at all levels to investigate the inclusivity of existing playgrounds.

### Rationale and Objectives

This research builds upon 4 previous systematic/scoping reviews that have examined inclusive/accessible playground design, focusing on the development of new playgrounds [21,22,25,29]. What these 4 studies do not offer is a tool to evaluate the inclusivity of *existing* playground structures. This is crucial knowledge for informing approaches to retrofitting playgrounds, intended to create supportive environments for children with disabilities to engage in unstructured outdoor play.

The purpose of this study is to explore *which tools exist to evaluate playground inclusivity to enable the participation of children with disabilities.* Playground inclusion will be operationalized as creating an environment where children have equal access to social and physical aspects of play, regardless of ability [25,30]. Unlike previous research, this study will narrow the breadth of evidence to examine literature that provides auditing tools to evaluate the design of existing playground structures. The identification, collation, and synthesis of tools to audit playground inclusivity have important implications for improving the participation and inclusion of *all* children in play opportunities. This review aims to inform evidence-based decision-making and the application of tools for practitioners (eg, government officials, child development and recreation practitioners, playground developers, and community disability champions) who are interested in evaluating local community playgrounds for inclusion.

## Methods

### Study Design

To explore available playground auditing tools, a scoping review will be conducted. Scoping reviews are a useful knowledge mobilization strategy to synthesize the heterogenous evidence available (ie, peer-reviewed research and grey literature), to determine gaps in knowledge, and to inform policy and practice [31,32]. The conduct of this study will adhere to the PRISMA-ScR (Preferred Reporting Items for Systematic Reviews and Meta-Analyses extension for Scoping Reviews) guidelines [33] and the study has been prospectively registered with the Open Science Framework (registration number: rycmj). Significant amendments to this scoping review protocol will be recorded in Open Science Framework.

### Search Strategy

The search strategy was developed in consultation with a Health Sciences Teaching and Learning Librarian at Western University and with all authors (consisting of experts in the fields of children’s health, physical activity, occupational therapy, disability/inclusion, geography, and planning). The primary author conducted an initial scan of the inclusive playground literature. It was determined that a variety of approaches have been used to audit playgrounds in both research and practice. Therefore, both peer-reviewed publications in scientific journals and grey literature (ie, government reports, research-informed articles, organizational publications) will be included in this review.

The primary search strategy will use electronic database searching, focusing on empirical research studies that employed quantitative and qualitative methods. The search strategy will include three key components: (1) the playground environment; (2) children with disabilities; and (3) audit tools for evaluating the inclusivity of the playground. The first two themes, environment and disability, will be searched with relevant keywords and Medical Subject Headings terms, combined using Boolean operators and adjusted for each database (see Multimedia Appendix 1 for the MEDLINE search strategy). The following databases will be searched: MEDLINE, Scopus, CINAHL, and Embase. Due to the plethora of terminology available to refer to audit tools (eg, toolkit, evaluation, audit, checklist, assessment), the third theme will be evaluated by hand during the screening process as a component of the inclusion/exclusion criteria. An audit tool will be considered broadly as any tool that can be employed to conduct an evaluation of the playground for inclusion of children with disabilities, using questions that can be completed by a playground auditor.

### Inclusion and Exclusion Criteria

Reference lists of all included studies will be hand-searched for additional articles and grey literature meeting inclusion criteria. Four previous systematic/scoping reviews examined inclusive/accessible playground design. Although these articles will not be included in the data analysis, the authors will screen the reference lists and articles/reports that have cited these reviews since publication, to identify additional eligible literature.

Peer-reviewed research identified through the search strategy, citation tracking, and hand searching will be screened according to the outlined inclusion criteria (Textbox 1) and exclusion criteria (Textbox 2).

**Inclusion criteria of peer-reviewed literature.**

1. The literature must be written in English or French.
2. The primary focus of the resource must evaluate the accessibility and inclusivity of existing playground structures.
3. The resource must include a tool to conduct an evaluation of the playground for inclusion of children with disabilities, using questions that can be completed by a playground auditor.
4. The resource focuses on any type of disability. Disability will be defined according to the *United Nations Convention on the Rights of Persons with Disabilities* [15]
5. The resource was published after 2000.

**Exclusion criteria of peer-reviewed literature.**

1. The full-text article cannot be obtained.
2. The “playground” is defined in an alternate context (eg, an environmental playground of bacteria [21]).
3. The focus of the paper is strictly on the epidemiology of injury or design playground safety [21].

Grey literature will be captured in this review to reflect the implementation of auditing tools in a practical context, across a variety of sectors/disciplines, internationally. To ensure a rigorous search method is employed, a three-step process for recording the relevant literature will be employed [34]. Step 1 will involve a search of a relevant grey-literature database, the Canadian Health Research Collection Database. Then, a targeted web-based Google search will be conducted. Finally, the reference lists of all peer-reviewed and grey literature included in the full-text screening stages will be examined for additional grey resources.

Grey literature must meet all inclusion and exclusion criteria specified for the peer-reviewed research (Textboxes 1 and 2). Acknowledging the potential volume of grey literature and tools available/used by individual organizations, an additional inclusion criterion will be placed on grey literature. To ensure that the results of this scoping review reflect best practices for end users, the grey literature resource must transparently report how a tool was developed. Two additional exclusion criteria will also be included: (1) grey literature that conducts secondary applications of tools with unjustified modifications to an original tool reported by another organization will not be included; and (2) examples of organizations applying existing tools in practice will not be included. In these situations, the primary source of the tools will be assessed for inclusion in this review.

It was determined that searches will be limited to work published from 2000 to present, to align with the search strategies of the 4 previous systematic/scoping reviews [21,22,25,29]. We expect that most peer-reviewed articles captured in this review will likely have been assessed in the 4 previous reviews examining playground design for inclusion/accessibility. Therefore, the search strategies were examined for consistency. Three of the reviews used search strategies that employed an inclusion timeline prior to 2000 [21,22,25], with a total of 8 articles included across the studies [35-41] (with one duplicated). To determine if the included articles were relevant to this study, they were screened by the primary author for their coherence with the eligibility criteria (Textboxes 1 and 2). No articles met the present inclusion parameters; therefore, the inclusion timeline of 2000 to present in this review is considered comprehensive. 

### Screening Process

Title and abstract screening of literature will be conducted by two independent researchers using the outlined eligibility criteria in Covidence [42]. Full-text records of the included peer-reviewed articles will be imported into Covidence for full-text screening by the same two reviewers. Any discrepancies will be discussed with a third reviewer until consensus is achieved. Full texts of the grey literature extracted from the 3-step process will be assessed by both screeners for inclusion, using Microsoft Excel. Literature carried forward from the full-text phase will be reviewed by all authors for consensus on inclusion/exclusion in this review. The results and study inclusion process will adhere to the “PRISMA 2020 flow diagram for new systematic reviews which included searches of databases, registers and other sources” [43].

### Data Extraction

Searches in all electronic databases will be run within the same week, to ensure data retrieval within the same time frame. Search results will be extracted to Covidence software [42], where they will be organized for the review phases (title/abstract screening, full-text screening, and data extraction). A search log to record the initial strategy and subsequent modifications (including reference list searching), and details on the identified studies, will be maintained by the primary author, in accordance with the PRISMA guidelines [33].

To adequately represent the findings from the peer-reviewed research and grey literature, two data extraction tables will be maintained. For each included article, data will be extracted using a targeted rule set presented in a standardized form. For the peer-reviewed research, the extracted data will include the following: (1) study details (ie, title, authors, year, country, design, purpose, participant details); (2) auditing tool details (ie, disability and environment factors assessed, tool used to conduct evaluation, measure of inclusion, questions used, components assessed, and psychometric properties); and (3) results of the study. This information will aim to contextualize the methodology for researchers considering conducting similar studies. For the grey literature, the extracted data will include the following: (1) resource details (ie, title, authors, year, country, resource type, purpose); (2) auditing tool details (ie, disability and environment factors assessed, measure of inclusion, tool used to conduct evaluation, questions used, rationale/evidence grounding tool, target audience/user group, components assessed, and psychometric properties); and (3) results of the playground audit (if applicable).

### Data Synthesis

The results will first summarize the main findings of this review including: (1) the screening process (from initial search to final selection of papers); (2) a descriptive numerical summary of the included studies; and (3) an outline of the audit tools used in research and practice. A synthesis of questions employed by the tools will be grouped thematically. The auditing tools will be summarized into 13 recommendations and one “promising practice” by Brown and colleagues [21], an evidence-based resource intended to provide guidance when designing new playgrounds with consideration to both the physical design and the surrounding built and social environments. Recommendations for future research and practice will be provided. All authors will be involved in this process.

## Results

**Figure 1:**
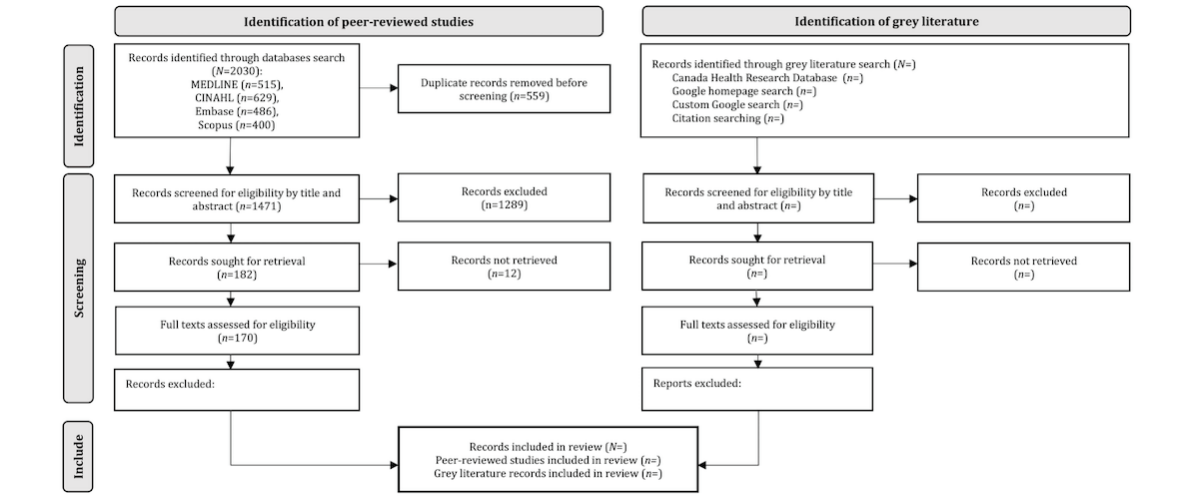
PRISMA flow diagram of findings to date. Inclusion criteria of white literature.

The electronic database searching for peer-reviewed literature was completed in December 2021. The search yielded 2030 results. All titles and abstract results were uploaded into Covidence [42]. There were 559 duplicate records removed in Covidence. Two independent reviewers screened the resulting 1471 records in Covidence, and 1289 were deemed irrelevant based on the eligibility criteria. As a result, the search produced 170 studies meeting the eligibility criteria. Figure 1 displays the PRISMA flow diagram representing findings available to date. Full-text findings in the peer-reviewed research will guide the grey literature review. The scoping review is planned for completion in 2022.

## Discussion

### Overview

This scoping review of both peer-reviewed studies and grey literature will be conducted to explore the tools that exist in research and practice to audit playgrounds for inclusivity, to enable the participation of children with disabilities. A synthesis of included articles will describe the auditing tools available and provide recommendations for researchers and stakeholders. Based on the preliminary results of this review, we hypothesize that our findings will demonstrate heterogeneity in the types and diversity of tools available. We will determine whether current resources are validated and evidence-informed, or if the generation of new resources should be considered.

### Evaluating for Inclusive Play on Existing Playgrounds

Play is considered a fundamental occupation of childhood and has important implications for children’s health and well-being [44-47]. Although playground structures are created with the intention to support play opportunities, children with a disability may experience barriers to engaging with these environmental structures [11]. This is an issue worth addressing as nearly 1 in 10 children worldwide have one or more disabilities [16]. Access to inclusive community playgrounds is crucial to provide health-supportive play opportunities for children of all abilities. To ensure all children can engage as equal participants despite disabilities, it is essential that researchers and practitioners engage in opportunities to evaluate community playgrounds [28].

Previous literature has examined methods for planning and developing new playgrounds for inclusion [21,22,25,29]. However, research has demonstrated that a clear, valid, and reliable strategy is needed to support opportunities for upgrading current community playgrounds to facilitate the inclusion of all children [48]. This review will expand on previous work by identifying, collating, and synthesizing tools available to audit existing playgrounds for inclusivity. Providing community stakeholders with the ability to evaluate existing playgrounds for inclusion is a crucial step in creating environments supportive of all children’s ability to participate in play, regardless of a disability. By synthesizing the results of this study to align with previous recommendations for building inclusive playgrounds [21], this review will identify tools that evaluate key opportunities for supporting and facilitating inclusion to guide evidence-informed decision-making, with the goal of future implementation of these tools in research and practice.

### Strengths and Limitations

The study offers an examination of tools to evaluate existing playground structures for inclusion and retrofitting, using a robust scoping review methodology to examine the extent of heterogeneous evidence available (ie, peer-reviewed and grey literature) to inform policy and practice, which are strengths of this work. However, there are limitations that must be acknowledged. Grey literature can be difficult to examine through typical systematic methods; therefore, we will attempt to minimize this potential limitation by using a research-informed strategy in consultation with a research librarian to limit discrepancies in locating and reporting these articles [34]. Furthermore, while psychometric testing of tools provided in the grey literature may be unclear or unavailable, we will attempt to mitigate this limitation by ensuring resources transparently report how a tool was developed and by drawing on their primary applications only. A final limitation to note is that language/culture bias may be present, as the articles will be limited to publications in English and French.

### Dissemination Plan

Engaging in intentionally planned and tailored knowledge translation activities is essential to sharing the findings of this review with key stakeholders, to inform evidence-based decision-making in research and practice. This will include community engagement sessions, conference presentations, executive summaries, and interactive recommendations for practice. This study makes an important contribution to the literature by systematically summarizing the available resources, bridging research with practice to offer tools and strategies to evaluate existing playground structures. This will aid in informing resource allocation for improving inclusivity for children with disabilities in their everyday environments.

## Appendix

Multimedia Appendix 1 MEDLINE search strategy.

## Abbreviations

PRISMA-ScR: Preferred Reporting Items for Systematic Reviews and Meta-Analyses extension for Scoping Reviews
