# Supplementary material for: Playground Inclusivity for Children With a Disability: Protocol for a Scoping Review
Source: JMIR Res Protoc. 2022 Jul 22;11(7):e37312. doi: 10.2196/37312 (PMC9356326; doi:10.2196/37312)
Supplement: Multimedia Appendix 1 [file resprot_v11i7e37312_app1.docx]

| **#** | **Searches** | **Results** |
| --- | --- | --- |
| 1 | inclusive.tw. | 20468 |
| 2 | inclusivity.tw. | 1086 |
| 3 | inclusion.tw. or Social Inclusion/ | 277944 |
| 4 | accessible.tw. | 91557 |
| 5 | accessibility.tw. | 49224 |
| 6 | adapt*.tw. | 609691 |
| 7 | equal*.tw. | 386247 |
| 8 | equit*.tw. | 31031 |
| 9 | universal design.tw. or Universal Design/ | 495 |
| 10 | disabled.tw. or Disabled Persons/ | 62852 |
| 11 | disabilit*.tw. or Intellectual Disability/ | 253821 |
| 12 | barrier free.tw. | 377 |
| 13 | 1 or 2 or 3 or 4 or 5 or 6 or 7 or 8 or 9 or 10 or 11 or 12 | 1683215 |
| 14 | playground*.tw. or Parks, Recreational/ | 3694 |
| 15 | playspace*.tw. | 6 |
| 16 | play space*.tw. | 125 |
| 17 | playscape*.tw. | 1 |
| 18 | play component*.tw. | 13 |
| 19 | play area*.tw. | 229 |
| 20 | play structure*.tw. | 33 |
| 21 | play park*.tw. | 3 |
| 22 | play environment*.tw. | 92 |
| 23 | play center*.tw. | 11 |
| 24 | play centre*.tw. | 8 |
| 25 | park equipment*.tw. | 3 |
| 26 | 14 or 15 or 16 or 17 or 18 or 19 or 20 or 21 or 22 or 23 or 24 or 25 | 4109 |
| 27 | 13 and 26 | 515 |
